# Partial response to niraparib in combination with tislelizumab in a patient with metastatic undifferentiated tonsillar carcinoma: a case report and literature review

**DOI:** 10.3389/fonc.2023.1078814

**Published:** 2023-05-18

**Authors:** Jing Zhang, Zi Dai, Pei Liao, Jieshan Guan

**Affiliations:** ^1^ Department of Oncology, The First Affiliated Hospital of Guangzhou University of Traditional Chinese Medicine, Guangzhou, China; ^2^ First Clinical Medical College, Guangzhou University of Traditional Chinese, Guangzhou, China

**Keywords:** metastatic undifferentiated tonsillar carcinoma, niraparib, PARP inhibitor, immune checkpoint inhibitors, next-generation sequencing, case report

## Abstract

Undifferentiated tonsillar carcinoma is an extremely rare head and neck cancer. The treatment options are challenging due to insensitivity to chemotherapy and easy development of drug resistance. In this study, we reported a case of advanced undifferentiated tonsillar carcinoma with multiple mediastinal lymph node metastases that failed to respond to chemotherapy. Next-generation sequencing (NGS) revealed germline BReast CAncer gene (BRCA) 1 mutation and a high tumor mutational burden. Poly (adenosine diphosphate [ADP]-ribose) polymerase (PARP) inhibitors have demonstrated efficacy in solid tumors with BRCA1/2 mutations. Immune checkpoint inhibitors (ICIs) provide a treatment option for unresectable head and neck cancer. After local control treatment by embolization, niraparib and tislelizumab were administered to this patient. A partial response (PR) was achieved, and progression-free survival (PFS) and overall survival (OS) were 12 months and 19 months, respectively. This case reveals molecular profiling as an important therapeutic strategy for rare malignancies with no standard of care. Moreover, the underlying synergistic antitumor activity of PARPi and PD-L1 blockade was reviewed.

## Introduction

Over the past 20 years, the incidence of primary tonsillar malignancies has increased at an annual rate of 0.35% (1). Squamous cell carcinoma with human papillomavirus (HPV) is the most common type with a good prognosis due to its sensitivity to radiation and chemotherapy (2–4). In contrast, HPV-negative undifferentiated tonsillar carcinoma is a rare malignancy, which is insensitive to chemotherapy. There is no available standard treatment for patients with inoperable or metastatic disease at diagnosis, and prognosis is poor. Programmed cell death 1 (PD-1) checkpoint inhibitors have been used in platinum-resistant recurrent/metastatic (R/M) head and neck squamous cell carcinoma (HNSCC), with an overall response rate of 14% in patients with PD-L1-positive/HPV-negative disease (5). Niraparib is an oral, highly selective poly (adenosine diphosphate [ADP]-ribose) polymerase (PARP)1 and PARP2 inhibitor with antitumor efficacy in BRCA1/2-mutated solid tumors (6). We present the first case of metastatic HPV-negative undifferentiated tonsillar carcinoma with germline BRCA1 mutation and high tumor mutational burden (TMB). The patient was administered niraparib and tislelizumab after failed chemotherapy and achieved a partial response (PR). With improvement in quality of life, the patient had a progression-free survival (PFS) of 12 months and an overall survival (OS) of 19 months. Moreover, we also systematically reviewed the literature and explored the potential mechanism of the synergy of PARP inhibitors and immunotherapy. The following case is presented in accordance with the CARE reporting checklist.

## Case presentation

**Figure 1:**
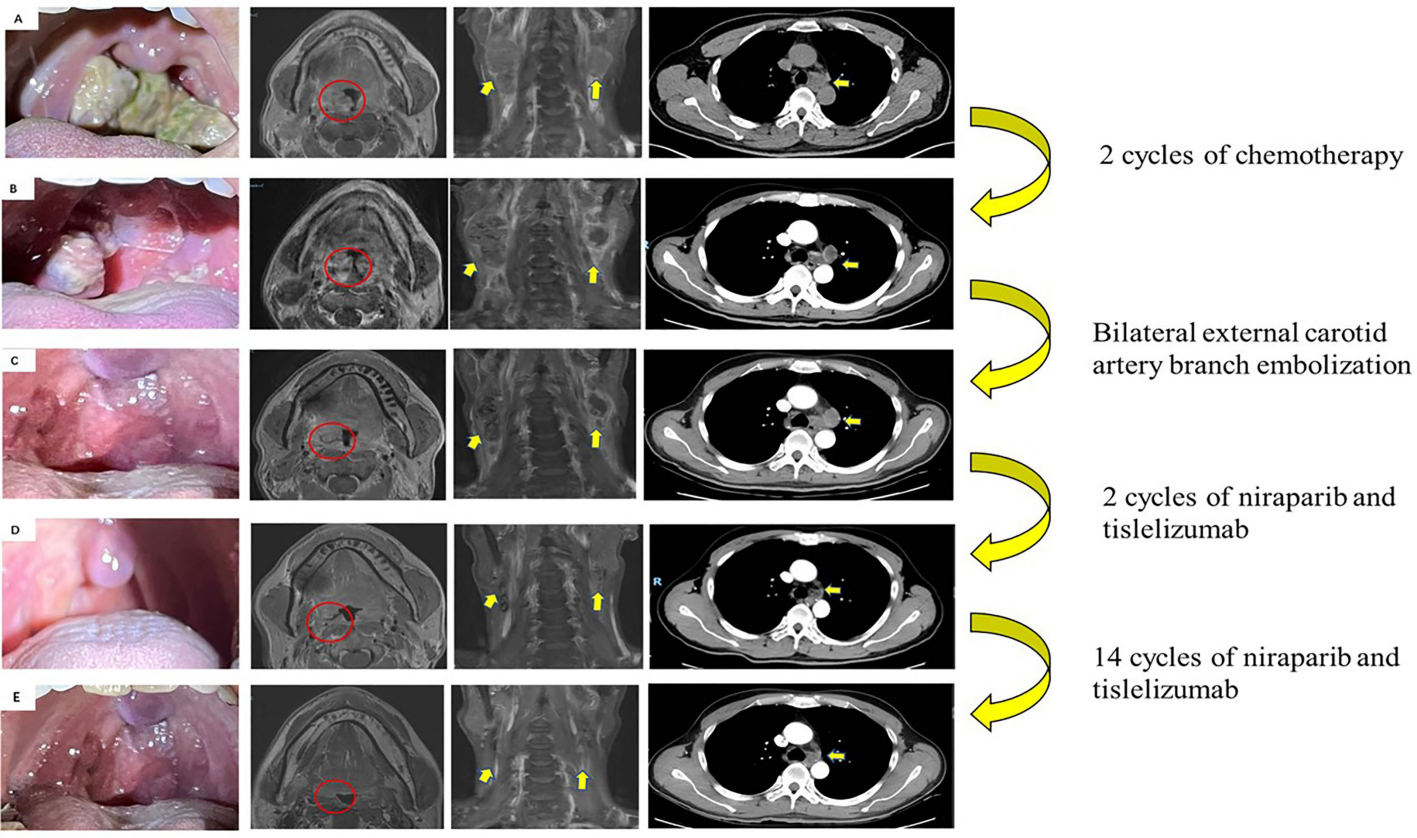
Images of primary tumor and metastatic lymph nodes during therapy. **(A)** Initial scans showed a mass in the right tonsil (red circle). Enlarged lymph nodes in the neck and mediastinum (yellow arrows) (December 2020). **(B)** Disease progression after 2 cycles of chemotherapy by RECIST criterion (February 2021). **(C)** After bilateral external carotid artery branch embolization (March 2021). **(D)** Partial response after 2 cycles of niraparib and tislelizumab by RECIST criterion (May 2021). **(E)** Re-examination of MRI and CT revealed no evidence of progression to the tonsils, cervical lymph nodes, and mediastinal and hilar lymph nodes (May 31, 2022).

**Figure 2:**
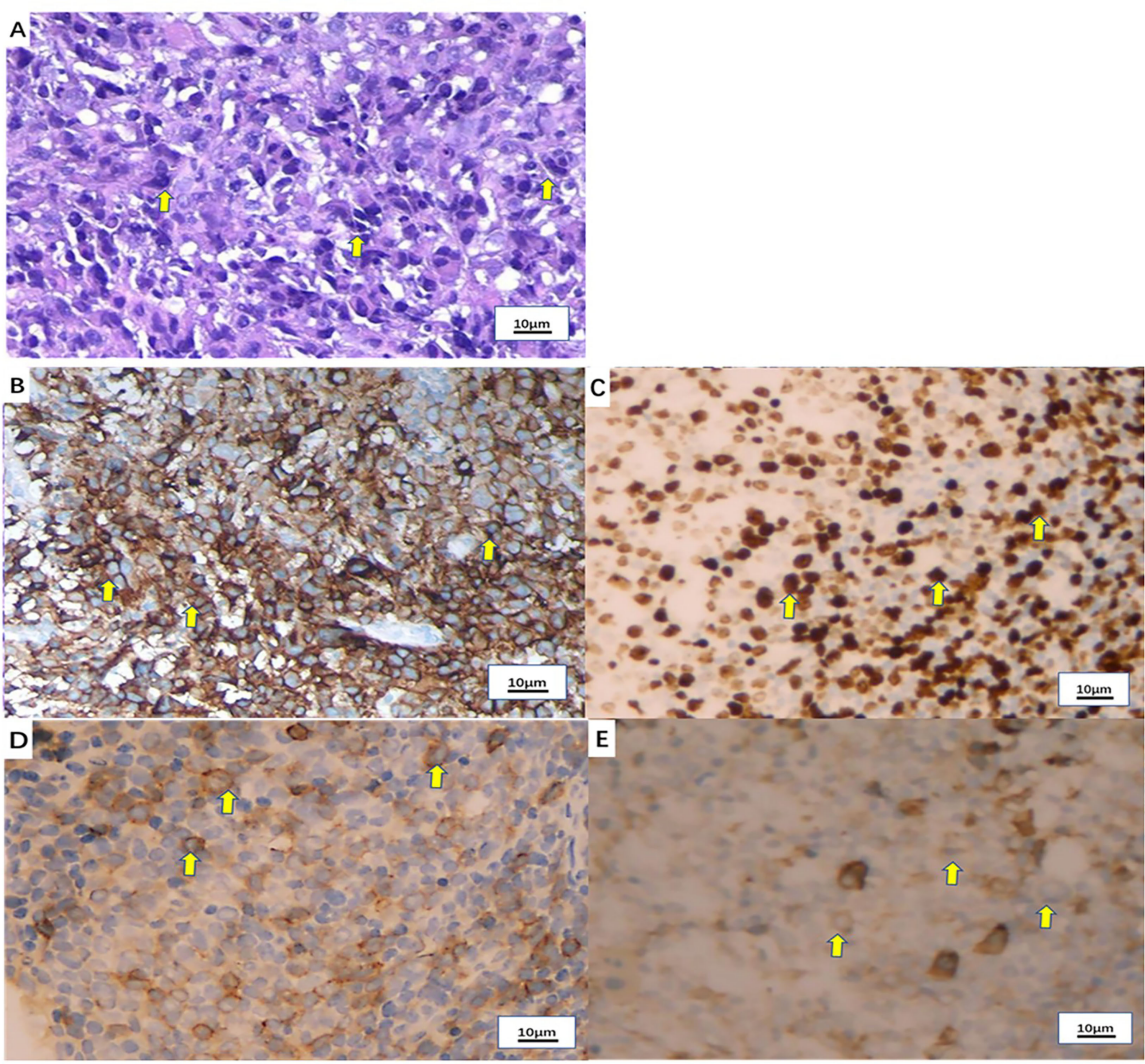
Pathological examination of the tonsil mass. **(A)** Hematoxylin-eosin (H&E) staining of the tonsil mass (x100). **(B)** PD-L1 expression by immunohistochemistry (90%+). **(C)** Ki-67 expression by immunohistochemistry (80%+). **(D)** CD138 expression by immunohistochemistry (partial +). **(E)** Vimentin expression by immunohistochemistry (+).

**Figure 3:**
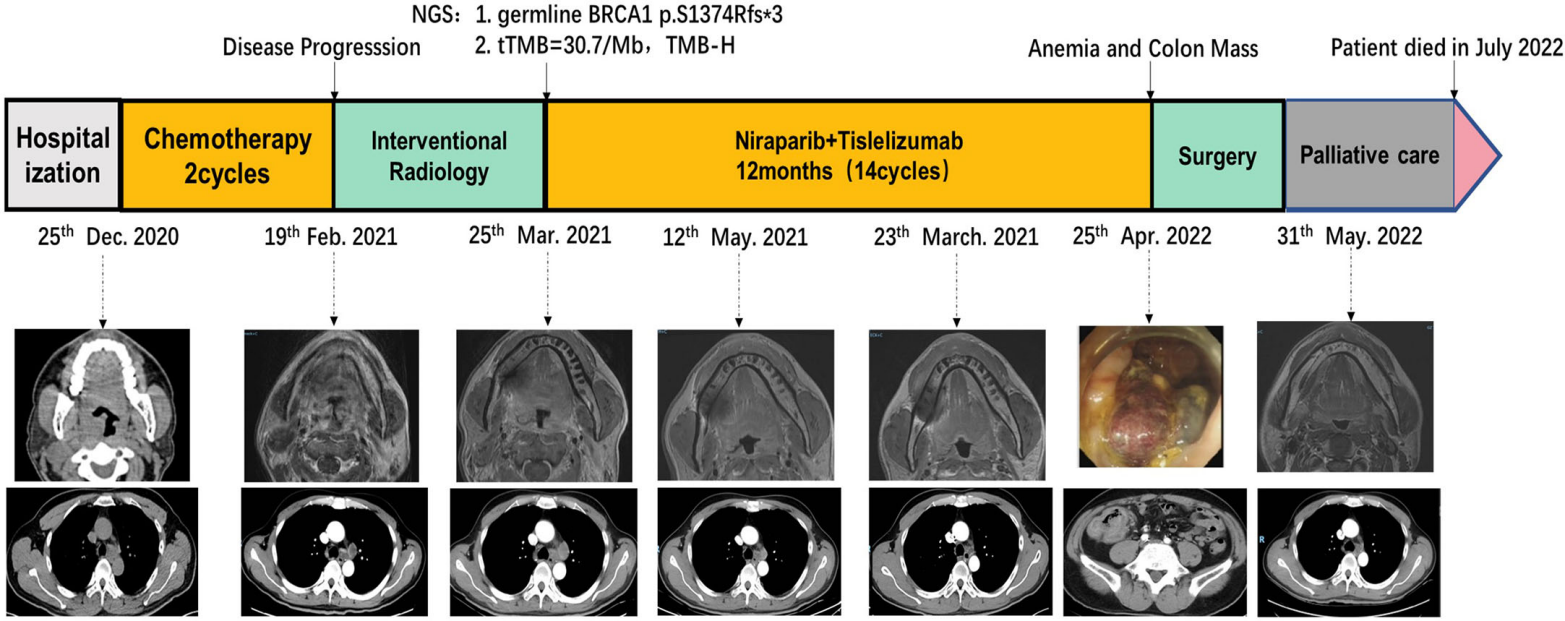
Treatment timeline.

**Figure 4:**
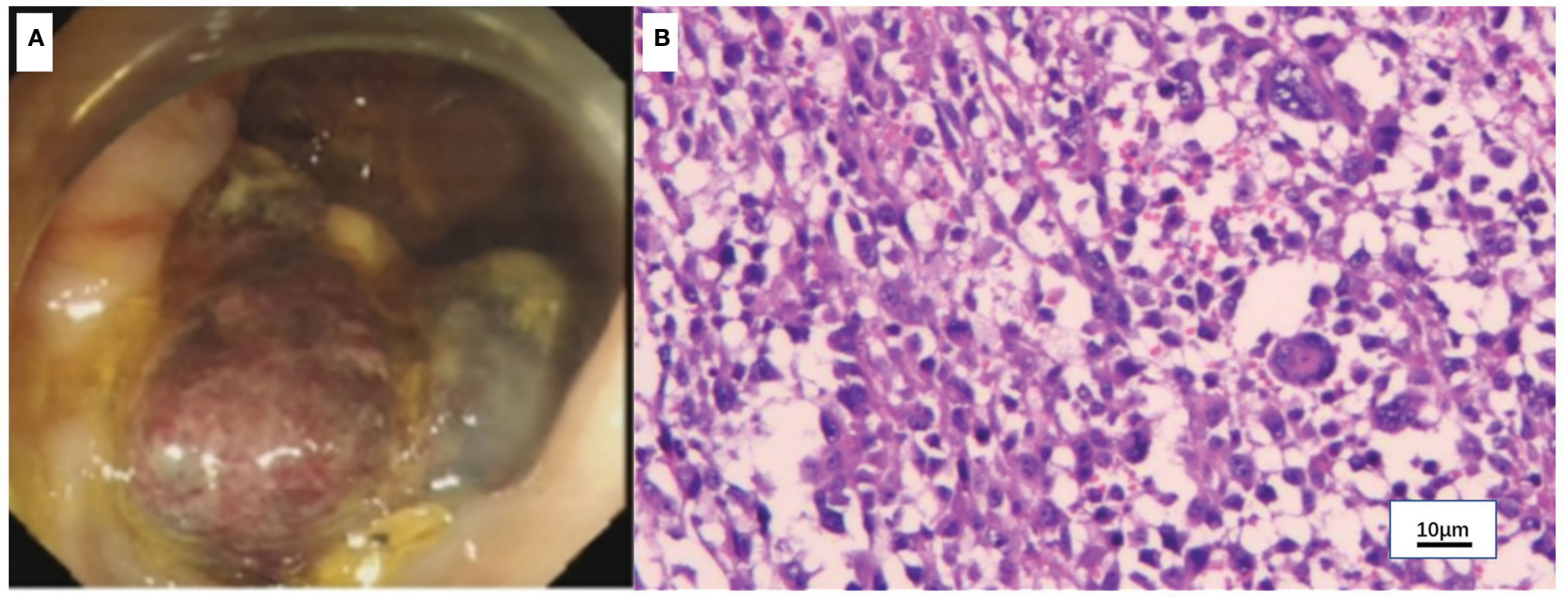
Colonoscopy and pathology of the colon mass. **(A)** Colonoscopy showed a mass in the ascending colon. **(B)** Hematoxylin-eosin (H&E) staining of the colon mass (x100).

A 50-year-old man with pharyngalgia and right neck mass presented to the hospital in December 2020. The patient had previously been diagnosed with hepatitis B infection and diabetes, had long-term work experience with chemical drugs and had no history of smoking, alcohol consumption and history of cancers. Physical examination showed an exophytic yellow-white cauliflower-like mass on the surface of the right tonsil, partially extending beyond the midline, but not invading other tissues around the oropharynx (**Figure 1A**). Several swollen lymph nodes were palpable on both sides of the neck. Head and neck magnetic resonance imaging (MRI) (December 20, 2020) showed a mass of 32 mm × 16 mm in the right tonsil and multiple enlarged lymph nodes around the bilateral carotid arteries. The largest lymph nodes were 40 mm × 23 mm (right) and 36 mm × 31 mm (left). Whole-body Positron Emission Tomography-Computed Tomography (PET/CT) (December 22, 2020) showed multiple enlarged lymph nodes in the mediastinum and hilum, with a maximum of 21 × 18 mm (**Figure 1A**). Biopsy of cervical lymph nodes and the tonsil mass revealed diffuse growth of medium to large lymphocyte tumor cells, indicating lymphoma and requiring further assessment. The biopsy specimens were delivered to the central laboratory for analysis (Guangzhou, China). Due to severe pharyngalgia, dysphagia and wheezing, the patient was administered two cycles of chemotherapy (doxorubicin liposomal at 40 mg on d1, vincristine at 2 mg on d1 and prednisone at 100 mg on d1-d5) from December 31, 2020. However, head and neck MRI (February 19, 2021) showed a 36 × 25 mm mass in the right tonsil, and the largest cervical lymph nodes were 45 × 37 mm (right) and 36 × 39 mm (left). Chest CT showed multiple enlarged lymph nodes in the mediastinum and hilus, with a maximum of 29 mm × 23 mm (**Figure 1B**). According to Response Evaluation Criteria in Solid Tumors (RECIST) version 1.1, the efficacy was evaluated as progressive disease (PD). The pathological results of the central laboratory were confirmed on March 5, 2021. Tonsil mass was demonstrated as malignancy by hematoxylin-eosin (H&E) staining which showed small foci of heterotypic tumor cells, some cells with light stained and obvious large nucleoli cell (**Figure 2A**). Immunohistochemistry (IHC) revealed that the tumor cells were positive for Ki-67 (95%), CD138 and Vimentin, PD-L1 (90%) (**Figure 2B–E**), and negative for CK, p16, CD20, CD3, CD30, CD4, CD8, LCA, CD5, CD7, ALK, CD56, CD31, ERG, CD21, CD23, CD38, CAM5.2, CD117, EMA, S-100, SOX-10 and Mum-1, κ, λ. Epstein-Barr encoding region (EBER) *in situ* hybridization was negative. The final diagnosis of this patient was based on a thorough review of gene rearrangements analysis, histopathology and IHC analysis. Lymphoma was excluded by the gene rearrangement assay (lymphoma biomarkers IGH, IGK and IGL were negative, S1). According to the H&E staining, IHC and PET/CT results, the patient was finally diagnosed with undifferentiated tonsillar carcinoma with multiple cervical and mediastinal lymph nodes metastases [cT2N2M1, stage IVC, American Joint Committee on Cancer (AJCC) 8^th^ edition]. Next generation sequencing (NGS, Geneseeq Technology Inc) identified germline BRCA1 gene P.S1374Rfs * 3 exon 12 frameshift mutation and a high TMB of 30.7 mutations/MB in the tumor tissue. Considering the high risk of operative hemorrhage and asphyxia, surgical oncologist believed that the risks of surgery outweigh the benefits. After carefully evaluating the patient’s performance status, treatment tolerance, tumor imaging and the feasibility of intravascular interventional therapy, the multidisciplinary team developed a focused treatment plan individualized to the patient. The patient received bilateral external carotid artery branch embolization on March 15, 2021 and March 18, 2021 because of the obvious enlargement of the tonsil mass and the high risk of airway obstruction. Intravascular interventional therapy was less invasive and had a low risk of surgical complications. After locoregional operation, the patient had only mild pain around the neck, the tonsil mass was reduced to 20×15 mm, and the largest cervical lymph nodes were 32 × 20 mm (right) and 27 × 17 mm (left) (March 25, 2021) (**Figure 1C**). According to the germline BRCA1 mutation, high TMB and the PD-L1 positive status, the patient was administered 300 mg niraparib QD and 200 mg tislelizumab Q3W from March 25, 2021. Follow-up MRI and CT (May 12, 2021) revealed a striking decrease in tumor burden after the second treatment cycle. The tonsil mass basically disappeared, and the largest cervical lymph nodes shrank to 21 × 18 mm (right) and 18 × 13 mm (left). The hilar lymph node was reduced to 20 × 14 mm (**Figure 1D**). The patient achieved a PR under treatment of niraparib combined with tislelizumab according to the RESISIT 1.1 criteria. Surveillance imaging showed continuous partial remission for 12 months (**Figure 3**). The patient developed mild fatigue, leukopenia (2.26×10^9^/L) and anemia (HGB 83g/L) in the first month of niraparib administration. These adverse events were rated as levels 1-2 (CTCAE 5.0) and resolved after niraparib was reduced to 200 mg QD. The patient resumed normal work with a high quality of life. In March 2022, the patient was admitted to the hospital with hematochezia and anemia (HGB 67 g/L). Colonoscopy revealed a mass in the ascending colon, accounting for about 2/3 of the intestinal lumen, with superficial fragility and hemorrhage (**Figure 4A**). Whole-body CT and barium meal examination of the digestive tract showed a mass of 43mm x 22mm in the proximal ascending colon, with no signs of new lesions elsewhere (April 25, 2022). The patient underwent ascending colectomy with lymph node dissection on April 28, 2022. Postoperative pathology showed that the colonic mass was metastatic undifferentiated tonsillar carcinoma, with no lymph node metastasis (**Figure 4B**). Re-examination of MRI and CT revealed no evidence of progression to the tonsils, cervical lymph nodes, and mediastinal and hilar lymph nodes (May 31, 2022) (**Figure 1E**). However, owing to the worse performance status after intestinal surgery, the patient declined any further treatment, except palliative care. Eventually, he died of multiple organ failure in July 2022. The patient’s PFS and OS were 12 months and 19 months, respectively, after treatment with niraparib and tislelizumab. The timeline of the relevant information is shown in **Figure 3**.

## Discussion

Malignant tumors of the tonsil account for about 1.3-5.0% of all malignant tumors in the whole body (2). The subtype of undifferentiated carcinoma is relatively rare and lacks data reports, especially HPV-negative tumors. A small sample size case series reported 16 cases of oropharyngeal undifferentiated carcinoma (7). Only one case was HPV-negative undifferentiated carcinoma, and the prognosis was much worse than that of HPV-positive patients. Moreover, patients with metastatic oropharynx carcinoma have a poor prognosis. Median PFS and OS in R/M HNSCC patients are about 5 months and 10 months, respectively (8). The HPV status and PD-L1 expression determine the treatment strategy. According to the WHO classification of head and neck tumors (2017), HPV-negative tumors are more genetically diverse and show a worse prognosis compared with HPV-positive tumors (9). The preferred therapeutics for R/M non-nasopharyngeal cancers are the PD-1 inhibitors including pembrolizumab and nivolumab (10–12). Unfortunately, current clinical trials were designed to recruit most common squamous-cell carcinoma cases, without including undifferentiated carcinoma. Moreover, immunotherapy acts selectively in the patient population, and only a minority of patients benefit from a monotherapeutic approach. The overall response rate remains quite low and disappointing. Only a 14% response rate was detected in PD-L1-positive/HPV-negative HNSCC (5). An estimated 85% of patients have no response to PD-1 inhibitors or have a response that is followed by disease progression and death. The final diagnosis of this patient was HPV-negative metastatic undifferentiated carcinoma of the tonsil. There are limited data from randomized, prospective studies to guide decisions regarding this rare type of head and neck cancer. The choice of therapy should be individualized based on patient characteristics. The search for new therapeutic strategies is of utmost importance in this case.

Through molecular profiling, targeted alteration was identified, and precision therapy was implemented. Given that the patient harbors a germline BRCA1 mutation, PARPi therapy could be considered. PARP1 and PARP2 are key enzymes detecting and repairing single strand DNA breaks (SSBs). PARP1/2 inhibition lead to an accumulation of SSBs and stalled replication forks, which subsequently lead to double strand breaks (DSBs) requiring homologous recombination repair (HRR). HRR function relies on proteins such as BRCA1 and 2. Since BRCA1/2 mutated cells are inefficient in HR, DSBs are repaired in an error-prone manner by nonhomologous end joining (NHEJ), inducing genomic instability that causes cell death (13, 14). Niraparib is a highly selective PARP1/2 inhibitor approved for maintenance therapy of advanced ovarian cancer (15). PD-1 inhibitor tislelizumab was also added to the treatment regimen due to high PD-L1 expression (90%) in the tumor tissue. PD-1 inhibitors have shown efficacy in head and neck cancer, especially in PD-L1-positive tumors (16). As a result, the patient had a long survival time with the combination of niraparib and tislelizumab after failed chemotherapy. The combination had an acceptable safety profile and favorable quality of life.

Using a literature review, we sought to summarize the rationale for the synergistic antitumor effects of PARPi and PD-L1 blockade. Evidence showed PARPi-induced genomic instability modulates the tumor microenvironment (TME) (17). PARPi promotes the accumulation of cytosolic DNA and increases TMB, then activates the cGAS-STING pathway, enhances T cell infiltration, and stimulates type I interferon expression, thereby priming the TME (18–20). In addition, DNA fragments within the cytoplasm induces the production and expression of neo-antigens on the cell surface, thereby increasing immune activation and the odds of ICI response (21). PARPi enhances immunosuppression by upregulating PD-L1 expression through the inactivation of GSK3β (22). Increased amounts of tumor infiltrating lymphocytes, neoantigen release, and PD-L1 upregulation driven by PARPi suggest an increase of immunogenicity and present a potential role for ICIs. The synergistic antitumor effects of PARP and PD-L1 blockade have been observed in different types of solid tumors (23). The TOPACIO study reported the efficacy of niraparib combined with pembrolizumab in BRCA-mutated recurrent ovarian and advanced triple-negative breast cancer (TNBC). In the intention to treat patients, an ORR of 25% and a DCR of 68% were recorded, while in BRCA-mutated tumors, the ORR and DCR were elevated to 45% and 73%, respectively (24). The JASPER study was the first to investigate the efficacy of niraparib plus pembrolizumab as a first-line treatment option for metastatic or locally advanced non-small cell lung cancer (NSCLC), and the antitumor activity of this combination regimen was confirmed in NSCLC. Patients with high PD-L1 expression (TPS ≥ 50%) achieved a favorable ORR of 56.3%, versus 44.8% for pembrolizumab monotherapy in KEYNOTE-024 (25). The MEDIOLA trial reported the efficacy of olaparib combined with durvalumab for advanced solid cancers, including TNBC, ovarian, cervical and uterine cancers. For patients with germline BRCA1/2 mutations, ORR and DCR at 12 weeks were 63% and 81%, respectively (26). In these trials, the most common toxicities included anemia, fatigue and thrombocytopenia, and immune-related toxicities were comparable with single agent PD-1 inhibitor therapy. These data are encouraging and suggest high efficacy and good tolerability for PARPi and PD-1 inhibitor in combination. Treatment-emergent hematologic events are most common in PARPi treatments. In our case, low-grade leukopenia and anemia were observed in the first month of niraparib administration. The dose of niraparib was modified from 300mg QD to 200mg QD to reduce hematologic toxicity. Immune-related toxicity was not observed in this case. Overall, niraparib combined with tislelizumab was well tolerated and complications were manageable. This combination regimen may benefit a broader population than monotherapy. This combination may be useful in other head and neck tumors with BRCA 1/2 mutations and needs to be validated by further studies.

## Conclusion

This study highlights the importance of molecular-matched therapy for rare malignancies with no standard of care. Targeted therapy based on genetic alterations detected by NGS may improve survival and the quality of life in these patients. The combination of PARPi with PD-1 inhibitor was effective and well-tolerated for the current patient because of BRCA mutation and high PD-L1 expression. Personalized therapy based on broad molecular profiling needs to be further explored.

## Data availability statement

The original contributions presented in the study are included in the article/supplementary material. Further inquiries can be directed to the corresponding author.

## Ethics statement

The studies involving human participants were reviewed and approved by Ethics Committee of the First Affiliated Hospital of Guangzhou University of Traditional Chinese Medicine. The patients/participants provided their written informed consent to participate in this study. Written informed consent was obtained from the individual(s) for the publication of any potentially identifiable images or data included in this article.

## Author contributions

JZ, ZD and PL collected the clinical, diagnostic and therapeutic information of the patient. JZ wrote and submitted the manuscript. JG revised the manuscript and identified the case. JZ, ZD and PL contributed equally to this work. All authors contributed to the article and approved the submitted version.
