# Supplementary material for: Partial response to niraparib in combination with tislelizumab in a patient with metastatic undifferentiated tonsillar carcinoma: a case report and literature review
Source: Front Oncol. 2023 May 18;13:1078814. doi: 10.3389/fonc.2023.1078814 (PMC10234503; doi:10.3389/fonc.2023.1078814)
Supplement: Supplementary file 1 [file DataSheet_1.pdf]

Supplementary 1.

中山大学肿瘤防治中心分子诊断科

华 南 肿 瘤 基 因 诊 断 中 心

基因分子诊断报告单

检测编号:2102235386-B

姓　　名:	性别: 男　年龄: 49岁	医院: 本院
门 诊 号:	病理号: 864371-B	科室: 病理科
临床诊断: 无	送检日期: 2021-02-24	医生: 黎敏
检测方法: 多重荧光PCR毛细管电泳法	检测仪器: ABI3500遗传分析仪	标本类型: 石蜡包埋组织

检测项目:　淋巴瘤基因重排

检测片段:

检测基因	片段	有效检测范围	结果
IGH	FR1-JH	310-360	-
	FR2-JH	250-295	-
	FR3-JH	100-170	-
	DH-JH	110-290. 90-420	-
	DH7-JH	100-130	-
IGK	Vk-JkΓ	120-160. 190-210. 260-300	-
	Vk-Kde+intron-Kde	210-250. 270-300. 350-390	-
IGL	Vλ-Jλ	140-165	-
TCRB	VB-JB	240-285	
	VB-JB	240-285	
	DB-JB	170-210. 285-325	
TCRG	VΓ1f、VΓ10-JΓ	145-255	
	VΓ9、VΓ11-JΓ	80-140. 160-220	
TCRD	Vδ+Dδ+Jδ	120-280	

检测结果: IGH 、IGK、IGL基因重排克隆性检测阴性。

（注: 该标本提取核酸质量片段化严重，不能完全排除假阴性可能，请结合临床综合考虑，必要时重新送检合格标本进行检[illegible]

检测者: 刘小云　　报告者: 何彩云　　报告日期: 2021-02-26

检测结果诠释:

采用BIOMED-2标准化基因重排克隆性分析系统，通过多重PCR技术检测免疫球蛋白（Ig）及T细胞抗原受体（TCR）克隆性基因重排。用于淋巴组织增殖性疾病的辅助诊断。最终诊断结果需结合形态学、免疫表型及临床。

联系方式: 中山大学肿瘤医院分子诊断科，电话: 020-87345687; 电子邮箱: fzzdk@sysucc.org.cn

地址: 广州市建设六马路青菜岗21号大院5号楼4楼; 邮编: 510060.

备注: 检测结果只对该检测标本负责，仅供临床医生参考。

Gene molecular diagnosis report from South China Cancer Gene Diagnosis Center

Test number: 2102235386-B
Date of inspection: 2021-02-24
Method: multiplex fluorescent PCR Capillary electrophoresis
Instrument: ABI3500 genetic analyzer

Gene	Segment	Detection range	Result
IGH	FR1-JH	310-360	-
	FR2-JH	250-295	-
	FR3-JH	100-170	-
	DH-JH	110-290,90-420	-
	DH7-JH	100-130	-
IGK	Vk-JkΓ	120-160,190-210,260-300	-
	Vk-Kde+intron-Kde	210-250,270-300,350-390	-
IGL	Vλ-Jλ	140-165	-
TCRB	VB-JB	240-285	
	VB-JB	240-285	
	DB-JB	170-210,285-325	
TCRG	VΓlf,VΓ10-JΓ	145-255	
	VΓ9,VΓ11-JΓ	80-140,160-220	
TCRD	Vδ+Dδ+Jδ	120-280	

Result：IGH, IGK and IGL gene rearrangements negative.
